# Effects of Multidisciplinary Rehabilitation Enhanced with Neuropsychological Treatment on Post-Acute SARS-CoV-2 Cognitive Impairment (Brain Fog): An Observational Study

**DOI:** 10.3390/brainsci13050791

**Published:** 2023-05-12

**Authors:** Paolo Rabaiotti, Chiara Ciracì, Davide Donelli, Carlotta Oggioni, Beatrice Rizzi, Federica Savi, Michele Antonelli, Matteo Rizzato, Luca Moderato, Valerio Brambilla, Valentina Ziveri, Lorenzo Brambilla, Matteo Bini, Antonio Nouvenne, Davide Lazzeroni

**Affiliations:** 1 Prevention and Rehabilitation Unit, Parma, IRCCS Fondazione Don Gnocchi, Piazzale Servi, 3, 43100 Parma, Italy; 2 Division of Cardiology, University Hospital of Parma, University of Parma, Viale Antonio Gramsci, 14, 43126 Parma, Italy; 3 Department of Public Health, AUSL-IRCCS of Reggio Emilia, Via Amendola, 42122 Reggio Emilia, Italy; 4 “Humandive”, Piazzale XX Settembre, 1/B, 33170 Pordenone, Italy; 5 Cardiology Department, “Guglielmo da Saliceto” Hospital, Via Taverna Giuseppe, 49, 29121 Piacenza, Italy; 6 IRCCS Fondazione Don Carlo Gnocchi, Via Carlo Girola, 30, 20162 Milano, Italy; 7 U.O. Medicina Interna di Continuità, Azienda Ospedaliero-Università di Parma, Via Gramsci, 14, 43126 Parma, Italy

**Keywords:** SARS-CoV-2, long COVID, brain fog, rehabilitation, neuropsychology, cognitive impairment

## Abstract

Concentration and memory impairment (named “brain fog”) represents a frequent and disabling neuropsychological sequela in post-acute COVID-19 syndrome (PACS) patients. The aim of this study was to assess whether neurocognitive function could improve after a multidisciplinary rehabilitation program enhanced with individualized neuropsychological treatment. A prospective monocentric registry of PACS patients consecutively admitted to our Rehabilitation Unit was created. The Montreal Cognitive Assessment (MoCA) was used to assess cognitive impairment at admission and discharge. A total of sixty-four (64) PACS patients, fifty-six (56) of them with brain fog, were treated with a day-by-day individualized psychological intervention of cognitive stimulation (45 min) on top of a standard in-hospital rehabilitation program. The mean duration of the acute-phase hospitalization was 55.8 ± 25.8 days and the mean in-hospital rehabilitation duration was 30 ± 10 days. The mean age of the patients was 67.3 ± 10.4 years, 66% of them were male, none had a previous diagnosis of dementia, and 66% of the entire sample had experienced severe COVID-19. At admission, only 12% of the patients had normal cognitive function, while 57% showed mild, 28% moderate, and 3% severe cognitive impairment. After psychological treatment, a significant improvement in the MoCA score was found (20.4 ± 5 vs. 24.7 ± 3.7; *p* < 0.0001) as a result of significant amelioration in the following domains: attention task (*p* = 0.014), abstract reasoning (*p* = 0.003), language repetition (*p* = 0.002), memory recall (*p* < 0.0001), orientation (*p* < 0.0001), and visuospatial abilities (*p* < 0.0001). Moreover, the improvement remained significant after multivariate analysis adjusted for several confounding factors. Finally, at discharge, 43% of the patients with cognitive impairment normalized their cognitive function, while 4.7% were discharged with residual moderate cognitive impairment. In conclusion, our study provides evidence of the effects of multidisciplinary rehabilitation enhanced with neuropsychological treatment on improvement in the cognitive function of post-acute COVID-19 patients.

## 1. Background

Patients recovering from a severe illness or after hospitalization due to SARS-CoV-2 infection (COVID-19) are more likely to report different prolonged symptoms or sequelae that persist for weeks to months after the acute disease [1,2]. The constellation of persistent symptoms after acute COVID-19 infection has been described by various terms, including “long COVID”, “post-COVID syndrome” (PACS), or “post-acute sequelae of SARS-CoV-2 infection” [3]. The prognostic impact of PACS is not well established, but recent evidence suggests a high rate of morbidity and mortality [4]. Among hospitalized patients recovering from a severe COVID-19 infection, fatigue, dyspnea, and myalgias are the most common PACS general symptoms. Moreover, attention, visuospatial abilities, orientation, and memory impairment (named “brain fog”) represent frequent and disabling neuropsychological sequelae in patients with PACS [5], and a positive correlation has been demonstrated between these symptoms and COVID-19 severity [6,7]. The mechanisms that lead to the development of neurocognitive impairment following acute SARS-CoV-2 infection are not well known; even though some studies hypothesize that they may be explained by different biological alterations (direct cytopathic action of the virus and inflammation), recent evidence seems to suggest a direct SARS-CoV-2 effect in areas implicated in memory, language, and visuospatial orientation, such as cingulate gyrus and the hippocampus. Moreover, recently, Fernandez et al. showed the abnormal activation of microglia in the subcortical white matter and hippocampus of mice affected by a mild form of COVID-19 [8]. More specifically, a reduction in oligodendrocytes precursor and oligodendrocyte in subcortical white matter, as well as a reduction in hippocampus neurogenesis, was observed [8]. The authors suggest a pivotal role of C-C motif chemokine 11 (CCL11), a chemokine associated with normal brain aging, in microglial dysfunction, similar to cognitive impairment syndrome due to H1N1 influenza and antineoplastic chemotherapy [8,9].

PACS brain fog has several implications for psychological and physical recovery after COVID-19, as well as in work reintegration. Since mild cognitive impairment is demonstrated as a marker of dementia development [10] (predictive marker), whether post-COVID-19 brain fog represents a marker of future neurocognitive decline is still an open issue. For these reasons, the early assessment and treatment of brain fog represents a challenge in post-COVID-19 evaluation.

This study aimed to assess whether neurocognitive function could improve after a multidisciplinary in-hospital rehabilitation program based on the physical and neuro-psychological treatment of patients with PACS characterized by neurocognitive impairment (brain fog) after acute moderate-to-severe SARS-CoV-2 infection that required hospitalization.

## 2. Methods

We created a monocentric prospective registry of PACS patients consecutively admitted to the Rehabilitation Unit of the “Don Gnocchi” Foundation of Parma (Italy) after discharge from the local hospital, where they had been treated for moderate-to-severe COVID-19. All patients were adults (aged 18+ years old), had been admitted to the sub-intensive or intensive care units (ICU) for COVID-19, and had developed PACS with or without cognitive impairment. Patients with no previous SARS-CoV-2 infection and whose symptoms were not ascribable to long COVID-19 were not considered for this study.

After hospital discharge with at least 2 consecutive negative SARS-CoV-2 swab tests, all patients completed a standard rehabilitation program, lasting approximately 30 days, consisting of supervised exercise sessions based on aerobic training lifestyle and risk factor management, counseling, and medical therapy optimization enhanced by a daily individualized neurocognitive rehabilitation program performed by resident psychologists. The 30-day program was administered to the patients on a daily basis, each session lasted around 45 min, and the entire rehabilitation was discontinued after its completion. The mental health therapists supervised the physical training sessions (the exercises were agreed with physiotherapists) and were responsible for administering the neurocognitive rehabilitation. The setting where the program was delivered was the Rehabilitation Unit of the “Don Gnocchi” Foundation of Parma (Italy).

The study was approved by the Ethics Committee on human research of the “IRCCS-Fondazione Don Carlo Gnocchi” (Italy) with the approval code 06_16/04/2020, released on 16 April 2020, and carried out following the Declaration of Helsinki after having obtained written informed consent from all the patients. 

At admission and discharge, anamnestic information, demographic data, clinical characteristics, laboratory markers, and neuropsychological data were collected. The Montreal Cognitive Assessment (MoCA) was used to evaluate cognitive impairment, the Impact of Event Scale-Revised (IES-R) was used to identify post-traumatic distress disorders [11], the Hospital Anxiety and Depression Scale (HADS) A and D were used to assess anxiety and depression levels [12], and the European Quality of Life (EuroQoL or, simply, EQoL) test was used to measure the patients’ quality of life. All of these outcomes were measured both at admission/baseline and at the end of the rehabilitation program.

### 2.1. Neurocognitive Evaluation and Rehabilitation Program

#### 2.1.1. Physiotherapy Rehabilitation Program

A standard, in-hospital rehabilitation program was performed with all subjects. Physiotherapy was different for every patient, depending on specific health conditions, in order to improve standing position (at admission), followed by mobilization of the upper body and proprioception, and then more difficult abilities (such as running or using/throwing a ball) with aerobic or mixed training. At admission, the patients were treated with exercises of trunk stability, bedside or in a sitting position. After one week of rehabilitation, the study participants with improvements in oxygen saturation and ability to keep a proper standing position underwent aerobic exercises and muscle strengthening to recover motor schemes. Different types of exercises were performed to improve muscular strength, mobilization of the upper body, and proprioception. Globally, the physiotherapy program was based on progressively active and aerobic physical activity, including both bodyweight and aided exercises performed with rehabilitation equipment (fitball, parallel bars, treadmill, exercise bike). The exercises were always supervised by the program therapists.

#### 2.1.2. Psychological Rehabilitation Program

According to our internal post-COVID-19 protocol, all patients underwent psychological evaluation with the IES-R, HADS, EQoL tests, and MoCA for a first-level cognitive screening able to discriminate and observe which cognitive areas were particularly deficient in the inpatient rehabilitation management. The MoCA test was preferred to the MMSA (Mini-Mental State Evaluation) since it was found to be able to investigate the areas more damaged by COVID-19 [13]. Cognitive impairment was classified according to the MoCA score as follows: normal (≥26 points), mild (18–25 points), moderate (10–17 points), and severe (<10 points) cognitive impairment.

After the initial screening, depending on the first-line tests, patients with cognitive impairment only performed an individualized psychological intervention specifically focused on improving the more damaged functions. In particular, a day-by-day individualized psychological intervention of cognitive stimulation (45 min) was performed in all patients with any degree of cognitive impairment (MoCA < 26) at admission. The different treatments were individualized and selected among the most deficient skills of the MoCA: visuospatial, executive, memory (verbal or visual, immediate or deferred, work), attentive (selective or sustained), or space–time orientation. 

The psychological intervention was also characterized by the enhancement of resilience skills, coping strategies, and a focus on the progressive changes in the patient’s rehabilitation needs. Moreover, further treatments were dedicated to the management of any anxious or depressive symptoms reactive to the disease, which was oriented to the recent past (experience of hospitalization) at admission and at discharge focused on the future (return to home). The psychological interventions were also focused on helping the patients improve their communication with family members. Finally, the same tests performed at admission were repeated on discharge. 

### 2.2. Statistical Analysis

Continuous variables were expressed as mean (M) ± standard deviation (SD), and categorical variables as a percentage (%). According to the MoCA score, patients were classified into four groups: normal (≥26 points), mild (from 18 to 25 points), moderate (from 10 to 17 points), and severe (<10 points) cognitive impairment (cut-offs for the definition of mild, moderate, and severe impairment are reported in brackets). Differences between MoCA severity (grading) were tested with the Pearson χ^2^ test. Cohen’s d was used to estimate the effective sample size (d = 1.11).

One-way repeated measures multivariate analysis of variance (MANOVA) was used to test any differences between admission and discharge MoCA data, as well as to determine any differences in MoCA scores after considering three different models. Model 1: adjustment for age/gender. Model 2: adjustment for baseline differences between groups (age, dyslipidemia, chronic obstructive pulmonary disease (COPD), previous cardiovascular disease). Model 3: adjustment for age, gender, in-hospital duration (days of acute COVID-19), the need for mechanical ventilation, and severity of COVID-19. 

Statistical significance was set at *p* < 0.05. All statistical analyses were performed with SPSS version 24 (IBM Corporation). 

## 3. Results

### 3.1. Baseline Characteristics

**Figure 1:**
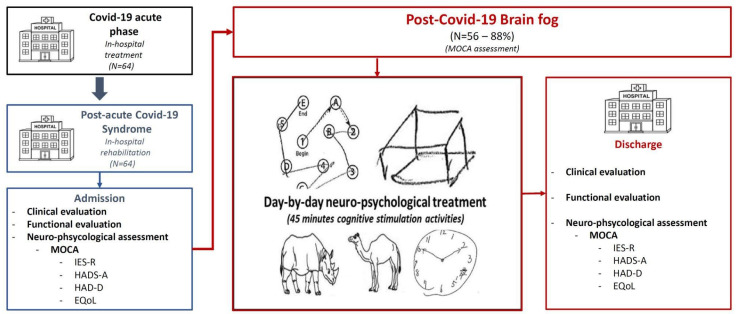
Study design. Figure description: 64 patients were admitted to our rehabilitation program after moderate-to-severe COVID-19; 88% of them showed neuropsychological impairment and were treated with day-by-day neuropsychological treatment. Cognitive evaluations were performed at admission and discharge.

A total of 64 consecutive PACS patients were admitted to our rehabilitation program after moderate-to-severe COVID-19 (Figure 1). At admission to the Rehabilitation Unit, the mean age was 67.3 (±10.4) years old, forty-two (65.6%) patients were male, thirteen patients (20.3%) had diabetes, forty-five (70.3%) had arterial hypertension, fourteen (21%) were active smokers, and seventeen (26.6%) had dyslipidemia (Table 1). Thirty-three patients (35.9%) had a history of cardiovascular disease, six (9.4%) of COPD, and four (6.3%) of chronic kidney disease (CKD). Fourteen patients (21.9%) were affected by gastrointestinal diseases, five (7.8%) by an oncologic disease, and nine (14.1%) had a prior history of thrombotic vascular problems. No subjects had any known cognitive impairment or dementia before the hospitalization. One patient had Parkinson’s disease without cognitive impairment and one subject had a history of previous cerebrovascular disease without cognitive impairment. Moreover, after the first psychological evaluation, attentive disorders without cognitive deficit were found in three subjects (2%), previous anxiety traits in twelve (8%), and previous depression traits in three subjects (2%). 

Significant differences were found between the MoCA groups regarding diastolic blood pressure (DBP), lower in patients with severe cognitive impairment (*p* = 0.014). No difference was found considering hospital duration, lung CT score, use of mechanical ventilation, hemoglobin concentration, C-Reactive Protein, D-Dimer level, or use of steroids, oxygen, or anticoagulants. All clinical, laboratory, and medical therapy data during hospitalization for the acute phase of COVID-19, as well as at admission of rehabilitation, are shown in Table 1.

The mean duration of the acute phase of COVID-19 hospitalization was 55.8 ± 25.8 (mean ± SD) days; 65.6% of patients had severe COVID-19 and 40 patients (63%) needed mechanical ventilation. During hospitalization, 54 (84.4%) patients had been treated with steroids, 10 (15.6%) with antivirals, 60 (93%) with low molecular weight heparin (LMWH), 48 (75%) with antibiotics, 24 (38%) with opioids, 18 (28%) with benzodiazepines (BDZ), and 18 (28.6%) with neuroleptic drugs. 

At admission to the treatment protocol, the mean MoCA score was 20.4 ± 5, 12% of the patients had normal cognitive function, and 57% showed mild, 28% moderate, and 3% severe cognitive impairment. Consequently, 88% of the patients showed a degree of cognitive impairment and were treated with a day-by-day individualized psychological intervention of cognitive stimulation. The remaining 12% of subjects with normal MoCA at admission underwent a standard rehabilitation program. No differences were identified between these groups considering gender, diabetes, hypertension, dyslipidemia, and active smoking. On the other hand, patients with more severe cognitive impairment showed higher age (*p* < 0.030) and a higher prevalence of previous history of cardiovascular disease (*p* = 0.021) and COPD (*p* < 0.001) (Table 2). 

Moreover, the Barthel index mean was 47.8 ± 18.4, the HADS-A mean score was 4.4 ± 3.3, the HADS-D score was 3.7 ± 3, and the IES-R score for post-traumatic distress means values was 16.6 ± 15.6.

### 3.2. Cognitive Evaluations between Admission and Discharge (Pre- and Post-Treatment)

**Figure 2:**
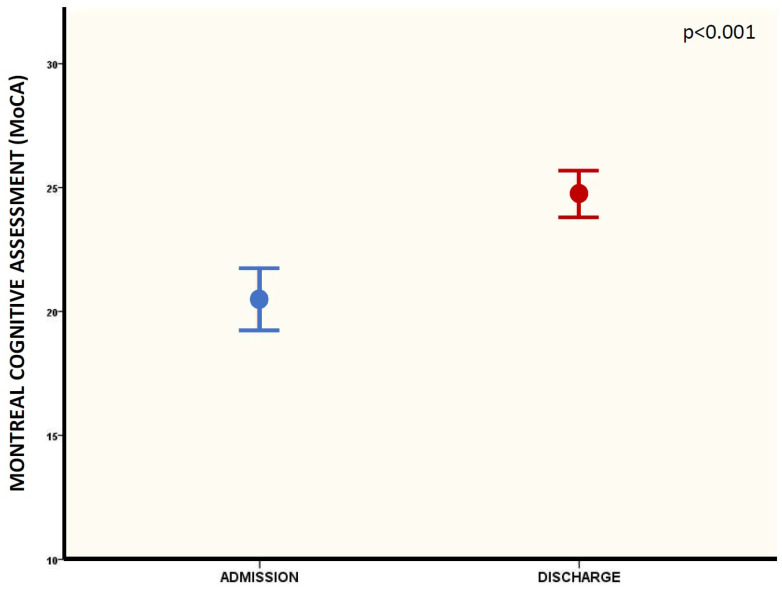
MoCA overall score at admission and discharge of rehabilitation (pre- and post-treatment).

**Figure 3:**
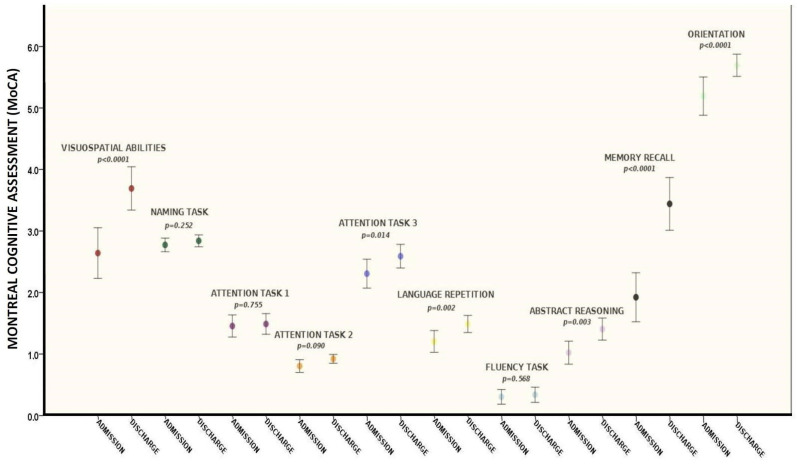
MoCA scores items at admission and discharge. Figure description: attention, orientation, visuospatial abilities, memory recall, abstract reasoning, and language repetition were significantly improved between admission and discharge of rehabilitation (pre- and post-treatment).

After the rehabilitation program (mean period 30 ± 10 days), the mean MoCA overall was 24.7 ± 3.7, corresponding to a significant improvement in cognitive function between admission and discharge (20.4 ± 5 vs. 24.7 ± 3.7—*p* < 0.0001, Figure 2) as a result of a significant improvement in the following domains: attention task (*p* = 0.014), language repetition (*p* = 0.002), memory recall (*p* < 0.0001), orientation (*p* < 0.0001), abstract reasoning (*p* = 0.003), and visuospatial abilities (*p* < 0.0001) (Table 3 and Figure 3).

**Figure 4:**
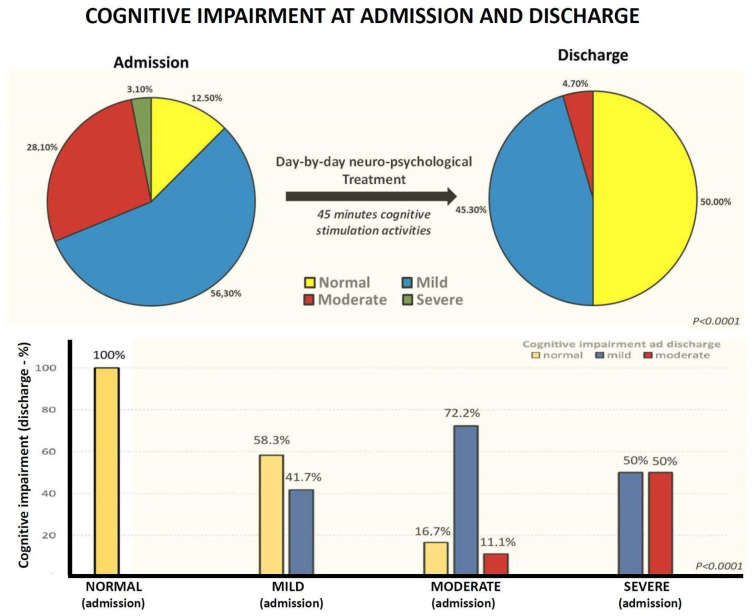
Upper side: percentages of MoCA levels (divided into four groups: normal, mild, moderate, and severe cognitive impairment) at admission and after the neuropsychological rehabilitation treatment. Lower side: percentage of cognitive impairment at discharge according to different groups at admission.

More specifically, at admission 12.5% of patients had no cognitive impairment, 56.3% showed mild cognitive impairment, 28.1% moderate cognitive impairment, and 3.1% had severe cognitive impairment, while at discharge half of patients (50%) were normal, 45.3% had mild cognitive impairment, 4.7% presented moderate cognitive impairment and none showed severe cognitive impairment (Figure 4). Moreover, 58.3% of patients with mild cognitive impairment at admission normalized their MoCA scores at discharge. A total of 16% of patients with moderate cognitive impairment at admission had normal MoCA, and 72.2% showed mild cognitive impairment at discharge. Of the two patients with severe cognitive impairment at admission (MoCA < 10), one had a mild cognitive impairment and the other showed a moderate impairment at discharge. No patient showed a worsening of cognitive impairment between admission and discharge from the rehabilitation unit (Figure 4). 

**Figure 5:**
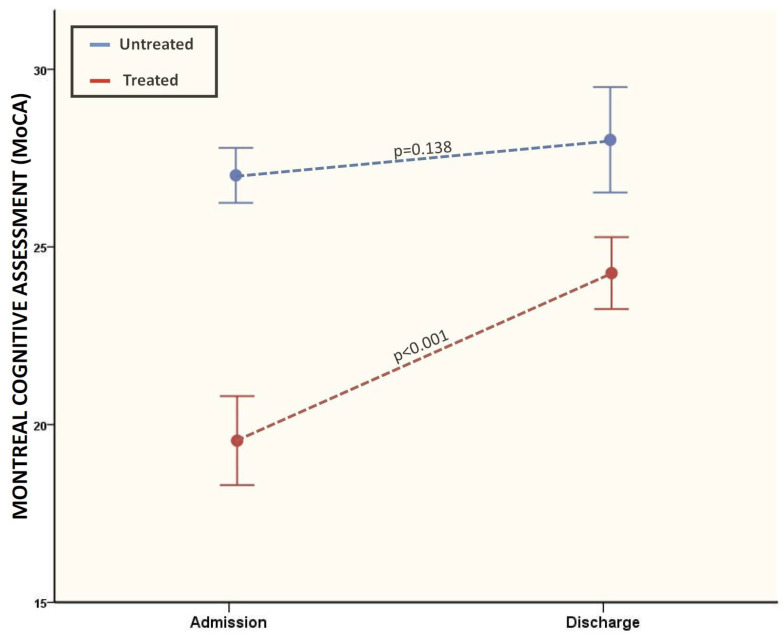
MoCA variations between treated and untreated patients from admission and discharge.

The MoCA improvement during the rehabilitation program was significantly higher if we considered only treated patients (19.5 ± 4.6 vs. 24.2 ± 3.7; *p* < 0.0001), while a lower non-significant improvement in MoCA scores was found considering untreated patients with normal MoCA scores at admission (27 ± 0.9 vs. 28 ± 1.7; *p* = 0.138) (Figure 5).

Moreover, MoCA score improvement remained significant after multivariate analysis including different models (Model 1, adjusted for age and gender, *p* = 0.003; Model 2, adjusted for age, dyslipidemia, COPD, and previous cardiovascular disease, *p* = 0.001; Model 3, adjusted for age, gender, days of acute COVID-19, the need of mechanical ventilation and severity of COVID-19, *p* = 0.007).

Finally, considering the other psychological tests, no significant improvement was found in the HADS-A, HADS-D, EQoL, and IES-R scores. Regarding the activities of daily living performance, a significant improvement in the Barthel index was observed from admission to discharge (*p* < 0.0001) (Table 4); however, no correlation between delta improvement in the MoCA and Barthel index (from admission to discharge) was found (r = 0.085; *p* = 0.417). 

## 4. Discussion

The aim of this study was to assess the effects of a multidisciplinary rehabilitation program, enhanced with a day-by-day neuropsychological treatment, in the neurocognitive capacity improvement of PACS patients with neurocognitive sequelae undergoing in-hospital rehabilitation after moderate-to-severe COVID-19. This was done by comparing neurocognitive function data, collected with the MoCA, at admission and discharge in 64 consecutive, non-selected PACS patients prospectively enrolled after the acute phase of COVID-19. Standard in-hospital rehabilitation was enhanced with a day-by-day individualized psychological intervention of cognitive stimulation in all patients with any degree of cognitive impairment.

The present study provides promising evidence of the positive effects of neuropsychological rehabilitation treatment in the cognitive function improvement of PACS patients with brain fog. More specifically, a multidisciplinary rehabilitation program resulted in a complete recovery of cognitive function in 42.8% of the patients with pre-treatment cognitive dysfunction. Additionally, 68% of the patients showed various degrees of improvement in cognitive function, and none showed a reduction in MoCA scores. However, at discharge, nearly half of PACS patients with brain fog still had mild (45.3%) or moderate (4.7%) dysfunction after a mean of 30 days of rehabilitation. Considering different specific MoCA items, cognitive recovery after treatment was mainly driven by a significant improvement in attention, language, memory, orientation, and visuospatial skills. Of note, MoCA improvement was independent of age, gender, COVID-19 severity, and days of hospitalization during the acute phase of the infectious disease.

PACS (Post-Acute COVID-19 Syndrome) is defined by persistent clinical signs and symptoms that appear while or after suffering COVID-19 (persistent after the acute phase of the disease and not explained by an alternative differential diagnosis) [1,6]. PACS is a heterogeneous condition that includes post-viral chronic fatigue syndrome, and sequelae in multiple organs, especially the cardiovascular system [14,15]. Regarding neurological sequelae, the most common are loss of memory or attention, visuospatial abilities, orientation, and language capacity impairment. PACS has been reported in most COVID-19 patients (from mild to severe COVID-19), irrespective of the severity of the symptoms of the acute phase [6]. Even if the etiopathogenesis of post-COVID-19 syndrome is largely unknown, several mechanisms have been postulated, probably related to multiple causes and pathogenesis pathways, including immune dysregulation, microbiota disruption, autoimmunity, clotting and endothelial abnormality, dysfunctional neurological signaling, and, ultimately, even genetic predisposition [16,17,18]. Although post-COVID-19 “brain fog” is still largely unknown, it represents a medical complication of large scientific interest [6,19,20]. In fact, a large amount of evidence has rapidly been produced and a significant spread of the brain fog phenomenon in patients previously infected with the new SARS-CoV-2 coronavirus all over the world has been demonstrated [1]. 

The mechanisms that lead to the development of neurocognitive impairment following acute SARS-CoV-2 infection are not yet well known, but some studies hypothesize that they may be multifactorial, due to both the direct cytopathic action of the virus and the inflammation produced in response to infection [21]. These factors would affect some brain areas including the cingulate gyrus and the hippocampus, areas dedicated to memory, language, and visuospatial orientation [5]. Recently, a study examined pre- and post-COVID-19 central nervous system imaging, including magnetic resonance imaging (MRI), computed tomography (CT), and positron emission tomography (PET) scan, in 441 patients with SARS-CoV-2 infection (of note, only 15 participants were admitted to hospital). Interestingly, in PACS subjects, authors found a significant thickness reduction in the gray matter of the entire cerebral cortex, more pronounced in the olfactory cortex, left parahippocampal gyrus, left orbitofrontal cortex, and temporal piriform cortex; areas strictly involved in memory, attention, orientation. These data suggest that modifications in the brain structure could be directly related (a consequence) to the subacute phase of COVID-19 [22]. Our data not only confirm an impairment in the executive (visuospatial) function of brain fog PACS patients, but also suggest that a day-by-day individualized psychological treatment improves cognitive functions, including a significant improvement in visuospatial/executive domains. In addition, our findings, showing a specific improvement in cognitive function areas related to memory, language, and visuospatial orientation, confirm the hypothesis that PACS brain fog represents a neurologic condition related to specific brain area impairment, rather than a merely multifactorial sequelae related to anxiety, depression or distress, as in other acute diseases requiring intensive care unit treatment. Despite a high percentage of patients with cognitive impairment, low levels of anxiety, depression, and distress were found among the patients enrolled in our study. In fact, similar research involving in-hospital patients with COVID-19 has demonstrated that levels of anxiety and depression measured with the HADS-A (4.9) and HADS-D (4.7) scores are, on average, higher than those reported in this study (4.4 and 3.7, respectively) [23]. In another study, with around 100 participants, it was suggested that one third of the subjects with recent COVID-19 had HADS-A and HADS-D scores higher than 8 [24], thus underscoring the need for assessing the patients’ psychological wellbeing after SARS-CoV-2 infection. Similar considerations can be drawn for distress symptoms measured with the IES-R, and recent studies have indicated average population scores of around 21 during the COVID-19 pandemic [25], higher than the mean of the patients analyzed in this study (Table 4). Apart from the MoCA and the Barthel Index, no significant pre–post changes were detected in the other scales sampled (EQoL, IES-R score, HADS-A, and HADS-D). Therefore, the program was well tolerated and did not worsen any of these psychological parameters. Additionally, a tendency towards amelioration was observed for the patient’s mood (HADS-D) and distress symptoms (IES-R) (Table 4), and it cannot be excluded that this tendency may become significant with a larger sample size or in patients with markedly impaired baseline mood and distress symptoms.

Moreover, although an improvement in functional capacity (Barthel Index) was also demonstrated, no correlation between MoCA improvement and Barthel improvement was detected, thereby suggesting that cognitive recovery was mainly related to psychological treatment instead of physical recovery. Similarly, since the improvement in cognitive function was higher only in the patients treated, it is plausible that cognitive recovery was primarily driven by cognitive stimulation treatment instead of physical therapy (which was performed in all subjects). 

In a study conducted on 87 patients admitted to the Rehabilitation Unit after a SARS-CoV-2 infection that required ICU or ordinary hospitalization, 80% of patients showed a degree of cognitive impairment, identified by the Mini-Mental State Evaluation (MMSE) and the MoCA tests [26]. The authors reported that cognitive impairment resulted mainly in deficits in short- and long-term memory, executive functions, abstraction, language, and orientation. Moreover, one month after discharge, cognitive impairment was reported in the vast majority of patients (ranging from 54% to 100% between different groups). More specifically, the percentage of residual cognitive impairment was higher in subjects who underwent non-invasive ventilation, with residual cognitive impairment in 83% [26]. Our findings show a similar rate of cognitive impairment at admission; on the other hand, we reported a lower residual cognitive impairment rate one month after rehabilitation in subjects undergoing neuro-psychological treatment, thereby suggesting an additive effect of this treatment in brain fog recovery on top of standard in-hospital rehabilitation care. Another study, examining 77 in-patients undergoing rehabilitation after the acute phase of COVID-19, showed an 80% cognitive impairment rate at admission with a mean MoCA score of 20.3 points; our data are in line with these findings. Moreover, at discharge, a significant improvement in the MoCA scores was reported in 45 patients only (60%) [27]. Of note, no psychological treatment was performed in previous studies on brain fog rehabilitation patients; in fact, only physical rehabilitation was performed.

The strengths of the present study are the following: first of all, a group of PACS patients underwent rehabilitation after moderate-to-severe COVID-19 (the infection severity was relatively homogeneous across the sample). Secondly, a prospective evaluation was carried out of the effect of neuro-cognitive treatment on top of the standard exercise-based physical program. Thirdly, there was a long day-by-day treatment period (30 days). Fourthly, a large battery of specific psychological evaluations was used to assess the level of cognitive impairment and other psychological items. Fifthly, the MoCA test is a validated international method for assessing cognitive impairment, and is more reliable than the MMSE in identifying which cognitive areas are more impaired. 

Several limitations should be highlighted, too. First of all, the sample size was small (64 patients); however, previous studies on PACS brain fog rehabilitation patients enrolled a similar number of patients. Secondly, a few patients had previous neurological diseases: one Parkinson’s disease, and one a previous cerebrovascular event. Thirdly, another limitation was the lack of a control group (non-rehabilitated patients and/or rehabilitated without psychological treatment), since all patients with MoCA impairment were treated; for that reason, we suggest considering as preliminary the data we reported on the comparison of MoCA improvement between treated and untreated subjects due to an evident treatment selection bias (only patients with cognitive impairment). Future randomized controlled trials are needed to clarify and confirm the efficacy of psychological treatment in PACS patients undergoing rehabilitation. 

In conclusion, although the clinical relevance of brain fog is still largely unknown, post-COVID-19 cognitive impairment has important implications in psychological and physical recovery after COVID-19, as well as in work reintegration. Moreover, since mild cognitive impairment is demonstrated as a marker of dementia development (predictive marker) [28,29], whether post-COVID-19 brain fog represents a marker of future neurocognitive decline is still an open issue. For these reasons, the identification and treatment of brain fog represents a challenge in post-COVID-19 evaluation. Our findings, showing a significant improvement in cognitive function during a multidisciplinary rehabilitation program enhanced with a day-by-day neuropsychological treatment, seem to suggest that both rehabilitations, as well as psychological intervention, could represent effective strategies to obtain PACS brain fog recovery.

## Figures and Tables

**Table 1:** Demographics, CV risk factors, and comorbidities data of the patients.

	Normal 8 (12.5)	Mild Cognitive Impairment 36 (56.3)	Moderate Cognitive Impairment 18 (28.1)	Severe Cognitive Impairment 2 (3.1)	*p*
Demographics					
Age (years), M (SD)	61.6 (12.9)	65.7 (8.8)	72.2 (10.8)	76.5 (2.1)	0.030 *
Gender (male), N (%)	0 (0)	12 (33.3)	9 (50)	1 (50)	0.095
CV risk factors					
Hypertension, N (%)	6 (75)	26 (72.2)	12 (66.6)	1 (50)	0.883
Dyslipidemia, N (%)	1 (12.5)	6 (16.7)	8 (44.4)	2 (100)	0.011 *
Active smokers, N (%)	0 (0)	10 (27.8)	3 (16.6)	1 (50)	0.110
Diabetes, N (%)	1 (12.5)	8 (22.3)	3 (16.6)	1 (50)	0.655
Comorbidities					
Cardiovascular, N (%)	0 (0)	12 (33.3)	9 (50)	2 (100)	0.021 *
COPD, N (%)	1 (12.5)	0 (0)	3 (16.6)	2 (100)	<0.001 *
Renal, N (%)	0 (0)	4 (11.1)	0 (0)	0 (0)	0.345
Neurological, N (%)	0 (0)	1 (2.8)	3 (16.6)	0 (0)	0.192
Psychiatric, N (%)	0 (0)	3 (8.3)	2 (11.1)	0 (0)	0.769
Gastrointestinal, N (%)	1 (12.5)	9 (25)	3 (16.6)	1 (50)	0.609
Oncological, N (%)	0 (0)	3 (8.3)	2 (11.1)	0 (0)	0.769

Legend: M, mean; SD, standard deviation; %, percentage; CV, cardiovascular; COPD, chronic obstructive pulmonary disease. * *p* < 0.05.

**Table 2:** Clinical, laboratory, and medical therapy data.

	Normal 8 (12.5)	Mild Cognitive Impairment 36 (56.3)	Moderate Cognitive Impairment 18 (28.1)	Severe Cognitive Impairment 2 (3.1)	*p*
Hospitalization (ACUTE PHASE)					
Days, M (SD)	72.3 (28.8)	53.9 (23.5)	54 (28.3)	40 (14.1)	0.232
Severe infection, N (%)	7 (10.9)	23 (35.9)	10 (15.6)	2 (3.1)	0.308
Mechanical ventilation, N (%)	7 (10.9)	21 (33)	11 (17)	1 (2)	0.393
Rehabilitation (POST-ACUTE PHASE) Admission Clinical data					
SBP (mmHg), M (SD)	123.5 (14.3)	125.5 (20.9)	132.6 (17.3)	100 (0)	0.134
DBP (mmHg), M (SD)	79.2 (13)	73.1 (8.1)	76.4 (10.5)	55.1 (7)	0.014 *
HR (bpm), M (SD)	80.1 (12.8)	80.6 (12.7)	89.61 (16.5)	72 (8.4)	0.092
SatO2 (%), M (SD)	97 (2.3)	96 (4.1)	96.6 (1.8)	99 (1.4)	0.617
O2 therapy, N (%)	4 (7.1)	28 (42.8)	23 (35.7)	4 (7.1)	0.783
Laboratory data					
Hb (g/dL), M (SD)	10.0 (3.76)	9.7 (4.1)	8.7 (5)	7.3 (8.9	0.752
WBC (×10^3^), M (SD)	6.8 (1.1)	7.1 (2.6)	9.3 (4.4)	7.8 (0.9)	0.111
RBC (×10^6^), M (SD)	3.7 (0.5)	3.9 (0.4)	3.9 (0.5)	3.7 (0.7)	0.693
RDW, M (SD)	14.7 (1.5)	16.3 (3.3)	15.5 (1.3)	15.7 (2)	0.480
PLT (×10^3^), M (SD)	282 (84)	270.5 (129.4)	270.7 (89.5)	224 (28.2)	0.936
Creatinine (mg/dL), M (SD)	0.66 (0.2)	0.68 (0.24)	0.62 (0.15)	0.41 (0.26)	0.367
CRP (mg/dL), M (SD)	1.6 (2.7)	2.4 (4.3)	2.4 (3.3)	3.1 (1.02)	0.944
ESR (mm/h), M (SD)	25	26.2 (20.5)	52 (27.2)	0 (0)	0.178
D-dimer, M (SD)	543	1006 (866)	517 (600)	0 (0)	0.549
COVID-19 therapy					
Steroids, N (%)	7 (10.9)	30 (46.9)	15 (23.4)	2 (3.1)	0.925
Beta blockers, N (%)	4 (6.2)	14 (21.8)	10 (15.6)	0 (0)	0.384
ACEi/ARBs, N (%)	1 (1.56)	7 (10.9)	3 (4.6)	1 (1.56)	0.671
ASA, N (%)	2 (3.1)	1 (1.56)	3 (4.6)	1 (1.56)	0.050
Antiviral, N (%)	1 (1.56)	6 (9.3)	3 (4.6)	0 (0)	0.925
LMWH, N (%)	8 (12.5)	37 (57.8)	16 (25.0)	2 (3.1)	0.192
Statins, N (%)	1 (1.56)	0 (0)	3 (4.6)	1 (1.56)	0.017 *
O2, N (%)	5 (7.14)	27 (42)	23 (35)	5 (7.14)	0.783
Antidepressant, N (%)	4 (6.2)	9 (14)	10 (16)	1 (2)	0.068
Opioids, N (%)	0 (0)	1 (2.8)	3 (16.7)	0 (0)	0.192
Neuroleptics and BDZ, N (%)	1 (12.5)	11 (30.6)	6 (33.3)	2 (100)	0.124
Antibiotics, N (%)	7 (10.9)	27 (42.1)	12 (18.7)	2 (3.1)	0.572

Legend: M, mean; SD, standard deviation; N, number; %, SBP, systolic blood pressure; DPB, diastolic blood pressure; HR, heart rate; Hb, hemoglobin; WBC, white blood cells; RBC, red blood cells; RDW, red cells distribution width; PLT, platelets; CRP, C-reactive protein; ESR, erythrocyte sedimentation rate; ASA, acetylsalicylic acid; BDZ, benzodiazepine. * *p* < 0.05.

**Table 3:** Improvement in MoCA overall and MoCa’s items.

	Admission, M (SD)	Discharge, M (SD)	*p*
MoCA overall	20.4 (5)	24.7 (3.7)	<0.0001 *
Attention task 1	1.4 (0.6)	1.4 (0.6)	0.755
Attention task 2	0.8 (0.4)	0.9 (0.2)	0.090
Attention task 3	2.3 (0.9)	2.5 (0.7)	0.014
Fluency task	0.3 (0.4)	0.33 (0.47)	0.568
Language repetition	1.2 (0.6)	1.4 (0.5)	0.002
Memory recall	1.9 (1.5)	3.4 (1.6)	<0.0001 *
Naming task	2.7 (0.4)	2.8 (0.3)	0.252
Orientation	5.1 (1.2)	5.6 (0.7)	<0.0001 *
Visuo-spatial abilities	2.6 (1.5)	3.6 (1.3)	<0.0001 *
Abstract reasoning	1.02 (0.7)	1.4 (0.7)	0.003

Legend: M, mean; SD, standard deviation. * *p* < 0.05.

**Table 4:** Neuro-cognitive and functional test at admission and discharge.

	Admission, M (SD)	Discharge, M (SD)	*p*
MoCA overall	20.4 (5)	24.7 (3.7)	<0.0001 *
EQoL	55.3 (21.1)	66.1 (19)	0.268
IES-R score	19.6 (15.6)	15.5 (11.7)	0.063
Barthel index	47.8 (18.4)	83.5 (19.4)	<0.0001 *
HADS-A	4.4 (3.3)	4.1 (3.3)	0.351
HADS-D	3.7 (3)	3 (2.7)	0.074

Legend: M, mean; SD, standard deviation. * *p* <0.05.

## Data Availability

All data are available by contacting the corresponding author.
